# N-Acetylcysteine as a novel rapidly acting anti-suicidal agent: A pilot naturalistic study in the emergency setting

**DOI:** 10.1371/journal.pone.0263149

**Published:** 2022-01-28

**Authors:** Davinder Hans, Anthony Rengel, Jaspreet Hans, Darryl Bassett, Sean Hood

**Affiliations:** School of Psychiatry and Clinical Neurosciences, The University of Western Australia, Perth, Western Australia, Australia; Chiba Daigaku, JAPAN

## Abstract

**Objective:**

N-acetylcysteine has a demonstrated role as an adjunctive therapy in psychotic and affective disorders as a treatment to reduce symptoms of Bipolar Affective Disorder, Major Depressive Disorder and Schizophrenia. However, its potential as a rapidly acting anti-suicidal agent has not yet been assessed. This naturalistic study evaluates its effect in thirty patients presenting following intentional medication overdose.

**Methods:**

Eighteen patients who ingested toxic doses of paracetamol received NAC whilst twelve other patients with other overdoses received standard supportive treatment in the emergency department setting. Symptoms were measured using the Montgomery-Asberg Depression Rating Scale and Clinical Global Impression scale at time of presentation, 24 hours, and seven days.

**Results:**

Baseline characteristics between groups were similar. Both groups showed a significant reduction in suicidality, as measured by the suicide item of the MADRS, over time (*p* < 0.001). However, there was a greater reduction in suicidality in the ‘NAC group’ compared to the ‘no-NAC group’ one-week post presentation (*p* = 0.014). A greater proportion of the ‘no-NAC group’ still exhibited severe depressive symptoms (MADRS >32) compared to the ‘NAC group’ (*p* = 0.044).

**Conclusion:**

This naturalistic study suggests NAC may have potential use as a rapidly acting treatment adjunct in major depressive disorder, warranting further investigation of its effects.

## Introduction

Depression is the most prevalent psychiatric disorder in Australia, with one in seven suffering from it throughout life [1]. The most devastating and final sequela of depression is suicide, which poses a serious burden on society. Unfortunately, the incidence of suicide is increasing and now represents the leading cause of mortality for people aged 15–44 [2, 3]. Although not every person who attempts suicide has a mental illness, the vast majority suffer from depression [2]. The lifetime risk of suicide within people with depression is estimated to be 6–15% [4].

Despite significant funding into prevention strategies, the evidence does not support the efficacy of most programs [5], nor has any individual strategy shown significant benefit over others [6]. Apart from containment of acute risk and optimizing treatment regime and social factors, the only traditional pharmacological treatments that have evidence for reducing suicidal ideation or suicide attempts are lithium and clozapine. Recent literature suggests lithium may result in up to a 20% reduction in suicidal ideation or attempts when compared to other agents [7, 8], as well as significant overall reduction in mortality [9]. Clozapine was first described as a legitimate anti-suicidal agent after the findings in the landmark InterSePT trial [10]. However, a recent meta-analysis suggests that there is inconsistency in findings, potentially due to a lack of randomisation in several studies [11]. Furthermore, clozapine and lithium are associated with a significant side effect profiles, which limit their use [12, 13].

Novel rapidly acting anti-depressants such as the N-Methyl-D-aspartate (NMDA) antagonist ketamine have demonstrated a significant sustained reduction in depressive symptoms and suicidal ideation in an acute setting [14]. However, its psychomimetic effects limit its safety and applicability within psychiatry. This has led to research into agents without psychomimetic properties and better tolerability profiles.

A preliminary study examined the feasibility of using single low dose intravenous ketamine acutely for 14 depressed patients with suicidal ideation in the emergency department [15]. They utilised the MADRS to assess symptoms and found that the symptoms decreased within 40 minutes of administration and did not recur during the subsequent 10 days. Despite the study’s limitations (including its small sample size), it tentatively suggested as a preliminary study that ketamine may be the first treatment other than electroconvulsive therapy (ECT) to produce rapid reduction of suicidal ideation. Subsequently, a series of randomised controlled trials, meta-analyses, and systematic reviews have provided further support for the effectiveness of ketamine for rapid reduction of suicidal ideation [16–20].

N-acetylcysteine (NAC) is a prodrug of cysteine that can be administered to humans both orally and intravenously. In addition to its most common clinical use, as a paracetamol antidote, it is also utilised in the management of COPD, contrast related nephropathy, and HIV [21]. There is an expanding body of evidence to suggest that NAC may have a role in the treatment of psychiatric conditions. We will discuss several properties of NAC in the following paragraphs.

### Oxidative damage and redox dysregulation

Oxidative damage and redox dysregulation appear to have important roles in the pathogenesis of psychiatric disorders due to the brain’s vulnerability to the toxic effects of oxygen free radicals [22]. NAC is believed to exert therapeutic antioxidant effects as a substrate for glutathione synthesis. Glutathione is a ubiquitous, intracellular antioxidant that maintains the oxidative balance in a cell and is able to neutralise reactive oxygen species via scavenging [23]. NAC readily crosses the blood brain barrier to increase intracellular glutathione levels by providing a cysteine substrate for glutathione synthesis in addition to acting directly as a scavenger of reactive oxygen species [23, 24].

### Regulation of glutaminergic neurotransmission

NAC also regulates glutaminergic pathways due to its provision of cysteine, which regulates the intra- and extra-cellular exchange of glutamate via the cysteine-glutamate antiporter [24]. This is of significance given that it has been purported that glutaminergic dysregulation has been identified as a potential pathological pathway in psychiatric disorders including depression and schizophrenia [25–27].

### Effects on the dopaminergic system

Effects of NAC on the dopaminergic system are significant, given that the dysregulation of dopamine is implicated in the aetiology of some psychiatric disorders. The regulation of the cysteine-glutamate antiporter can also regulate the release of dopamine from the pre-synaptic membrane [28]. The aforementioned anti-oxidant effect of NAC can also negate excessive dopamine which can cause neurotoxicity due to oxidative stress [29].

### Anti-inflammatory properties of NAC

Finally, NAC may have a therapeutic role in psychiatric disorders because of its anti-inflammatory properties [30]. It has been demonstrated that dysregulated inflammatory pathways are noted in people who are depressed [31], and these pathways are likely to affect the production of neurotransmitters and contribute to the pathology of the disorder. NAC has been shown to reduce inflammatory cytokines in addition to its effect on oxidative stress, highlighting its potential use in depression and other psychiatric disorders [32].

### NAC–role in psychiatry

Several studies have examined the clinical implications of NAC. A series of double blinded placebo-controlled studies of individuals with Bipolar Affective Disorder, Major Depressive Disorder and Schizophrenia have shown significant improvements in symptoms over time. A meta-analysis of seven studies in schizophrenia showed that when NAC was administered, a significant reduction in both positive and negative symptoms was seen, particularly if administered for longer than 24 weeks [27]. In mood disorders, these studies have shown a significant improvement in core symptoms including a greater incidence of remission [33–37].

A 12 week double-blinded, randomised, placebo-controlled study of individuals (n = 252) with a current episode of Major Depressive Disorder (MDD) provided limited evidence for NAC as an adjunctive therapy in unipolar depression [38]. Interestingly, response and remission was significantly greater in the NAC group at 16 weeks [38]. In addition, the rate of reduction of symptom severity from baseline to week 16 was significantly higher in the NAC group, suggesting potential use of NAC as a rapid-acting adjunct. Finally, participants with the more severe baseline depressive symptoms (MADRS score ≥25) in the NAC group had significantly lower CGI-S score than those in the placebo group at weeks 6, 8, 12 and 16, suggesting that adjunctive NAC may have greater benefit in the treatment of more severe depression [38].

The primary significance of our study is the potential efficacy of NAC as a rapid acting therapy in treating acute suicidality, a clinical condition for which rapid onset treatment is needed. Our naturalistic study examines this potential with acutely suicidal patients within the emergency department setting.

## Methods

### Design

**Fig 1:**
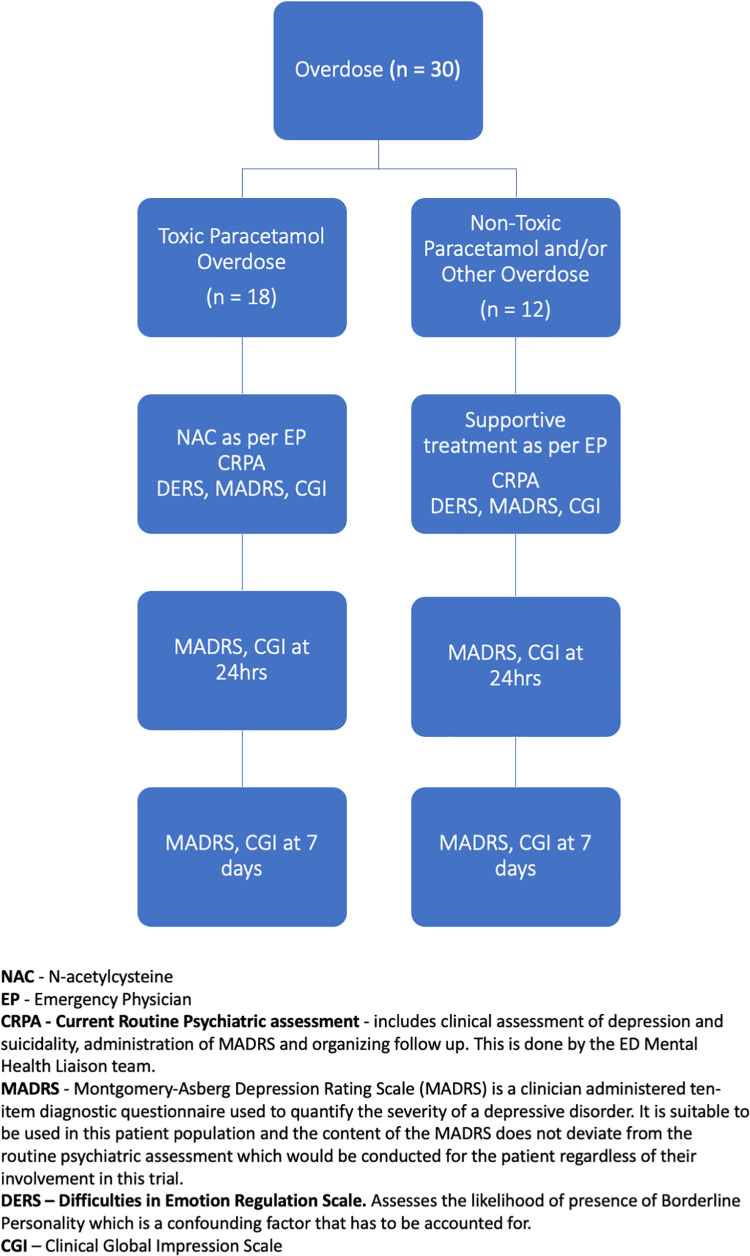
Study design and management of patients presenting to ED following intentional overdose.

Approval was obtained from the Ethics committee (HREC Reference Number 2014–050). This naturalistic study recruited thirty patients who presented to a tertiary hospital Emergency Department in Perth, Western Australia following an intentional medication overdose. As per standard practice, patients who had ingested toxic doses of paracetamol according to the Mathew-Ruckman nomogram were given weight-based doses (100mg/kg) of N-acetylcysteine (NAC) as directed by the Emergency Department (ED) physicians [39]. Eighteen patients were identified with toxic paracetamol overdoses and received NAC whilst the remaining twelve received supportive treatment for their overdose and did not require NAC (as they did not have a toxic paracetamol overdose). No treatment was given or influenced by the investigators. The study compared the psychometric results for group at initial interview, one day and seven days later, as summarized in Fig 1. We note that a self-report measure, the Difficulties in Emotion Regulation Scale (DERS), was also administered as part of routine practice. We did not include this patient-administered scale in analyses, because it was not consistently completed and there was a large amount of missing data.

### Measures

The Montgomery-Asberg Depression Scale [MADRS; [4040] is a 10-item clinician-rated scale that assesses the core symptoms and cognitive features of clinical depression, rating them on an ordinal scale from zero to six. In this study, the overall MADRS score was used as the primary measure of depressive symptoms, and MADRS Item 10 was used as a primary measure to rate suicide. Whilst no specific cut-off value was used to measure efficacy, the number of patients with severe depression (MADRS ≥ 32) at presentation and post was assessed.

The Clinical Global Impression Scale is a subjective measure used by mental health practitioners to assess severity of illness and response to treatment. It is a seven-point scale where a score of 1 represents great improvement in severity, 4 is neutral and 7 is significant decline. It was used as an adjunct to the MADRS in this study to track subjective changes in the patient’s mental state.

### Psychiatric assessment

Patients (regardless of participation) who presented with a drug overdose underwent a Current Psychiatric Risk Assessment (CPRA), which consisted of a one hour interview, the completion of the Mini International Neuropsychiatric Interview (MINI-5) to establish the diagnosis of a Major Depressive Episode (DSM-IV criteria), administration of the MADRS, Clinical Global Impression (CGI) scale, an area Health Service based Brief Risk Assessment (BRA) assessing static and dynamic risk factors of suicidality and rating it out of 28 points with high risk being above 14, and disposition planning. The CPRA was undertaken by a trained mental health practitioner, who identified suitable patients for study inclusion.

Patients were reviewed 24 hours after the initial interview or following commencement of NAC treatment. Patients were contacted seven days later either face to face (if they had a hospital admission within the same tertiary hospital) or via telephone. At both these time points, the MADRS and CGI were readministered, as seen in Fig 1.

### Selection criteria

To meet criteria for inclusion in the current study, all participants must have been at least 16 years of age, able to provide consent and must have taken an intentional overdose. They must have also suffered from a Major Depressive episode (mild, moderate, or severe) as determined by the MINI-5. For participants under 18 years of age, if they were living independently and were determined to be a mature minor [according to the legal principle of Gillick competence; [41, 42], informed consent was able to be granted by the participant following the CPRA, otherwise it must have been granted by the participant’s parents or guardians.

Participants were deemed ineligible if they were under 16 years old, were unable to give consent due to sedation, intellectual disability, being medically unwell, or being unable to speak English. In addition, participants were deemed ineligible if they were determined during the CPRA to have significant cognitive impairment, delirium, distress, or agitation, as well as very severe suicidality (with a corresponding BRA suicidality score > 18, as requested by the Ethics Committee).

Of note, there was significant variability in the baseline pharmacological therapy each patient was receiving at the time of enrolment. Patients were not deemed ineligible for the study on this basis. However, patients who required changes to their psychopharmacotherapy (due to a suicidality score > 18) were not included in the studied sample to reduce any confounding pharmacological effect.

Participants who were suitable and agreeable to inclusion in the study signed a consent form in line with the National Statement on Ethical Conduct in Human Research (NHMRC) guidelines. Participants were welcome to withdraw from the study at any time.

### Statistical analysis

For each time point in both treatment arms, the MADRS total score and CGI were used in the statistical analysis. In addition, item ten of the MADRS (MADRS10), rating suicidal thoughts, was separately analysed. The distributions for each outcome were examined with normality established for the MADRS Total score through distributional plots, Q-plots and the Shapiro-Wilk Test. Means and standard deviations were used to describe the MADRS total score. Median scores and ranges were used to describe the MADRS10 and CGI. Box plots were produced to visually examine the change over time for each group.

Repeated measures ANOVA determined between and within group differences for MADRS total score, with parametric post hoc tests carried out to see where the differences were. Contrasts and their 95% confidence intervals were produced. The MADRS Total score was further assessed by dichotomising the outcome into >32 and ≤32. The differences between the groups at time points 1 (24 hours after the overdose and for those in the ‘NAC group’, after NAC infusion) and 2 (seven days after the overdose) were assessed using a difference in proportions test.

MADRS10 and CGI were analysed using non-parametric methods due to their unipolar skewed distributions. Friedman’s test was used for within group differences, which was followed up with Wilcoxon signed rank test if the overall test demonstrated a p value <0.05. As baseline differences for MADRS10 and CGI were present, this was determined for everyone for time point 1 (T1-T0) and time point 2 (T2-T0). Between group differences were assessed using the Mann Whitney U test comparing baseline scores to time points 1 and 2.

All data was analysed using Stata 14.1 (StataCorp, College Station, TX). Statistical significance was considered p<0.05.

## Results

18 subjects had a paracetamol overdose requiring NAC (‘NAC group’) whilst 12 subjects did not require NAC (‘no-NAC group’). Patient characteristics are reported in Table 1. There was no significant sex distribution between groups however female patients made up 70% of the overall study cohort. The age distribution for each group was not significantly different.

**Table 1:** Patient characteristics.

		**NAC n (%)**	**no-NAC n (%)**
**Gender**	Male	5 (27.8)	4 (33.3)
	Female	13 (72.2)	8 (66.7)
		**mean (SD)**	**mean (SD)**
**Age**		36.1 (12.9) range 19–60	35.2 (13.6) range 21–61

### Between group differences

**Fig 2:**
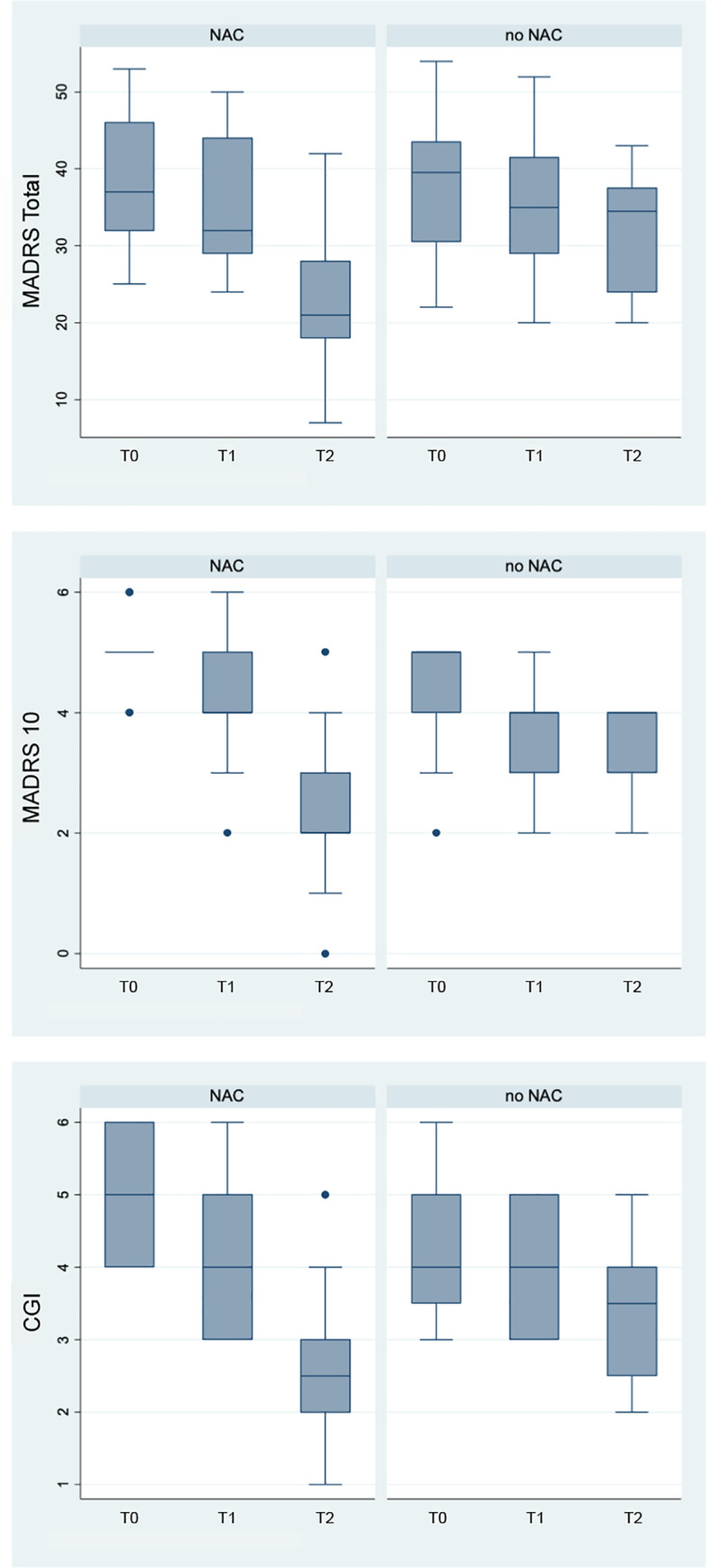
Between group differences for the ‘NAC group’ and ‘no-NAC group’. Plots show median, range, and first and third quartile for scores on the MADRS total scale, MADRS 10, and CGI at baseline, 24 hours post overdose, and one week post overdose (T0, T1, and T2, respectively) for each group.

Between group differences are presented in Table 2. At baseline there was no difference between the groups for MADRS total (difference = -0.94, p = 0.781). For MADRS 10 and CGI there appeared to be a difference between the groups at baseline. Although the median MADRS 10 was the same (median = 5), the ‘NAC group’ had a range of scores between 4 and 6, whereas the ‘no-NAC group’ ranged from 2 to 5. However, it is noted that this difference did not meet statistical significance (p = 0.064) but given the influence of the small sample size on p values the suggested differences between the groups needs to be taken into consideration. CGI was different between the two groups at baseline with median scores of 5 vs 4 (NAC vs no-NAC, p = 0.047). Fig 2 displays the differences between the groups for each of the outcome measures and demonstrates there are baseline differences for MADRS 10 and CGI.

**Table 2:** MADRS >32 –between group differences.

Outcome Variable	Group	Proportion MADRS >32 at each time point n (%)			Between Group Differences	
		T0	T1	T2	T1	T2
MADRS Total >32	NAC	14 (77.8)	9 (50.0)	4 (22.2)	0.179	0.044
	no-NAC	9 (75.0)	9 (75.0)	7 (58.3)		

At T1 there were no statistical differences between the two groups. However, at T2 there was a difference between groups on each of the outcome measures, with the ‘NAC group’ demonstrating lower scores (all ps <0.05)–see Table 2. For MADRS total score a significant difference was noted between the groups at T2, with a contrast of 8.55 (95% CI: 1.81 to 15.30, p = 0.014), indicating a lower score in the ‘NAC group’ and an overall significant reduction in depressive symptoms.

There was a significant difference between the groups for T2 for the proportion of subjects with a MADRS score greater than 32 and severe depressive symptoms. At T2 only 22% of the ‘NAC group’ had a score >32, vs 58% in the ‘no-NAC’ group.

### Within group changes

Within group changes are presented in Table 3. Both groups changed over time for all measures with a trend of reducing scores over time. The one clear exception was for the ‘no-NAC group’ from T1 to T2 where no change appeared to take place with the median MADRS10 score remaining as 4 (p = 0.317). In addition, the score changes for MADRS total and CGI for the ‘no-NAC group’ from T0 to T1 were small and did not reach statistical significance. There were more substantial changes in scores for the ‘NAC group’ for all outcome measures from T1 to T2. Specifically, the MADRS total score changed from 35.61 to 23.44 over this time period (p<0.001), which indicates a statistically significant reduction in depressive symptoms.

**Table 3:** Outcome measures at each time point for each group–within and between group differences.

**Outcome Variable**	**Group**	**Outcome Measures at each time point mean (SD)**			**Within Group Differences contrast (95% CI)**			**Between Group Differences contrast (95% CI)**	
		**T0**	**T1**	**T2**	**T1 vs T0**	**T2 vs T0**	**T1 vs T2**	**T1**	**T2**
**MADRS Total**	**NAC**	38.61 (8.91)	35.61 (8.87)	23.44 (9.56)	-3.0 (-5.36, -0.64) p = 0.013	-15.17 (-17.53, -12.80) p<0.001	-12.17 (-14.53, -9.80) p<0.001	-0.44 (-7.9, 6.30) p = 0.869	8.56 (1.81, 15.30) p = 0.014
	**no-NAC**	37.67 (9.66)	35.17 (9.21)	32.00 (8.32)	-2.50 (-5.39, 0.39) p = 0.090	5.67 (-8.56, -2.77) p<0.001	3.17 (-6.06, -0.27) p = 0.032		
**Outcome Variable**	**Group**	**Outcome Measures at each time point median (range)**			**Within Group Differences p value**			**Between Group Differences p value**	
		**T0**	**T1**	**T2**	**T1 vs T0**	**T2 vs T0**	**T1vs T2**	**T1**	**T2**
**MADRS item 10**	**NAC**	5 (4–6)	4 (2–6)	2 (0–5)	0.002	0.001	0.001	0.646	0.001
	**no-NAC**	5 (2–5)	4 (2–5)	4 (2–4)	0.006	0.002	0.317		
**CGI**	**NAC**	5 (4–6)	4 (3–6)	2.5 (1–5)	0.002	0.001	0.001	0.138	0.001
	**no-NAC**	4 (3–6)	4 (3–5)	3.5 (2–5)	0.084	0.005	0.014		

## Discussion

Our study provides promising initial evidence to suggest that NAC may have a rapidly acting anti-suicidal and antidepressant effect on patients who have taken a purposeful overdose of a toxic substance with suicidal intent in Major Depressive Disorder. Whilst the results demonstrate that regardless of administration of NAC, there was a trend towards reduced severity of illness and suicidality, the ‘NAC group’ showed a significant reduction in symptoms within 24 hours from baseline, which was not shown in the ‘no-NAC group’ until one week post administration. Comparing the two groups however, whilst no significant difference existed at 24 hours, there was a significant difference one week post NAC.

Despite not showing a statistically significant difference in symptom reduction compared to the ‘no-NAC group’ at 24 hours, the results support the theory that NAC may work as an anti-suicidal agent and as adjuvant therapy in depression. This is further supported by the greater proportion of patients in the ‘NAC group’ who were considered to have a ‘non-severe’ depression at one week (defined by MADRS < 32). It is noteworthy, that the measures of symptoms are consistent with this change in the signs of depressive illness.

Whilst we considered our selection criteria reasonably robust, our samples were quite heterogeneous. As per the naturalistic study design, there were multiple confounders in this study, and due to the small sample size, statistical control of these confounders was not possible. The main confounders in this study were the presence of a Personality Disorder, the presence of other psychiatric illnesses, the treatment regimen of patients (both in the ‘NAC’ and ‘no-NAC’ group) and the presence of other comorbid physical health diseases.

The main comorbid psychiatric illness present in subjects was an Anxiety Spectrum disorder, including Generalised Anxiety Disorder, Social Anxiety, and Panic Disorder. This was present in ten of the subjects and had preceded the onset of the Major Depressive episode. The presence of physical health problems was not consistently noted in the CRPA. Physical health issues pose as confounders because poorer physical health is typically associated with poorer mental health outcomes [43]. Of note, there was no difference in the approach to treatment in patients who had had a paracetamol overdose compared to patient who did not have a paracetamol overdose, except for the administration of NAC.

In both groups, there was significant heterogeneity in terms of baseline psychopharmacotherapy. Whilst we were required to exclude patients with severe suicidality as per the Ethics committee requirement and hence mandated changes to their standard therapy to improve symptomatic control, it is difficult in a naturalistic study to account for any interactions that may have occurred between NAC and their standard therapy. The limit to the intensity of suicidal thinking of our subjects also limits the generalisability of the observed effects, as the effect of NAC may vary with the severity of such ideation.

Finally, we must acknowledge the role of non-pharmacological measures such as counselling and provision of social supports that may have on influence mood and psychometric outcomes. Whilst no formal psychotherapy was initiated during the study period, the provision of additional psychosocial support has been shown to provide significant benefit to patients in crisis [e.g. [44]. However, considering this is standard of care it would be unethical to withhold such support in this study.

This is the first study to assess NAC in the role as a rapidly-acting antidepressant and further explores its potential use as an adjuvant therapy in Major Depressive Disorder. Further studies with greater controls and larger samples will be needed to confirm our effect and determine NAC’s role as an augmentation and/or anti-suicidal agent, and confirm the time course of its effects. We suggest that a randomised, blinded placebo control trial performed in the emergency setting to assess NAC’s anti-suicidal effects would be valuable. Further research should also investigate the role of comorbidities, and the effect of NAC on suicidality in different patient groups, such as people with Bipolar Affective Disorder. Whilst our study purposely considered overdose, a future study design should consider all presentations for suicidality, with adjustments made during analysis for cofounders including medication type and comorbid psychiatric disorders. In particular, an analysis using a multi-factor model [45] may reveal subtle effects in particular symptom domains not seen in our study.

## Conclusion

This study provides preliminary, naturalistic evidence that NAC could have an anti-suicidal and antidepressant effect in patients with Major Depressive Disorder who have attempted suicide with an intentional pharmacological overdose. We propose that it is possible that NAC may alter glutamate metabolism sufficiently to provide a rapid antidepressant effect with a subsequent sustained anti-suicidal benefit for up to one week and suggest that a randomised controlled trial is warranted to formally investigate this possibility.

## Supporting information

S1 Data (CSV)
